# Supplementary material for: Variability of the lymphocyte-to-monocyte ratio in patients with chronic kidney disease on hemodialysis
Source: PLoS One. 2025 Oct 31;20(10):e0333523. doi: 10.1371/journal.pone.0333523 (PMC12578246; doi:10.1371/journal.pone.0333523)
Supplement: S2 File — Pilot study presented as scientific poster. Results presented at the 2019 Congress of the Latin American Society of Nephrology and Hypertension (https://slanh.net/). Content available in Spanish. (PDF) [file pone.0333523.s002.pdf]

ESTUDIO COMPARATIVO DE PARÁMETROS HEMATOLÓGICOS Y BIOQUÍMICOS DE PACIENTES CON Y SIN DIABETES MELLITUS SOMETIDOS A HEMODIÁLISIS. EXPERIENCIA DE UN CENTRO PRIVADO DE DIÁLISIS AMBULATORIA, LIMA- PERÚ

P_SAB289

Díaz-Obregón D.1,2,Goyoneche-Linares G.1,2, Castañeda-Torrico M.1, Castro-Núñez M.1,
Liendo-Liendo C.3, Pacheco-NapuriA.3,Arrunátegui-Correa V.1,4,5

Daysi Díaz O.
E-mail: daysidi22@yahoo.es

[1]ONG InnovaCare. 2EDUPYME. 3CENESA.4Biomedical Technologies.5Facultad de Medicina, Universidad San Martin de Porres.

La *diabetes mellitus tipo 2* (DM2) y la enfermedad renal crónica (ERC) son problemas de salud pública a nivel global, por el rápido incremento de sus prevalencias y la carga de enfermedad y los costos sanitarios que generan. Ambas patologías producen un estado de inflamación severa y crónica con alteraciones inmunitarias que se agudizan durante el tratamiento de hemodiálisis (HD), por lo que es necesario mejorar la estratificacióndelriesgodeestospacientesenlabúsquedadefactorespredictivosde gravedady/oprogresiónde laenfermedad.

OBJETIVO

- Comparar los parámetros hematológicos y bioquímicos de pacientes con y sin DM2 sometidos a HD en un centro nefrológico privadoduranteelaño 2018.
- Identificar si existe relación entre el ratio linfocito/monocito (LMR) en pacientes con y sin DM2, sometidos a HD en un centro nefrológicoprivadoduranteelaño 2018.

MATERIALES Y MÉTODOS

Estudio analítico retrospectivo para analizar parámetros hematológicos y bioquímicos de pacientes diabéticos con ERC sometidos a HD y compararlos con pacientes no diabéticos del centro nefrológico CENESA durante el año 2018. La población fue de 92 pacientes, de los cuales los pacientes con DM2 que cumplieron los criterios de selección fueron 11 (edad promedio 68.2 ± 7.95 años), comparado con un grupo de 22 pacientes sin DM2 (edad promedio 68.1 ± 9.76 años).

Para el análisis estadístico se aplicó un modelo de regresión lineal para analizar la relación del LMR con la enfermedad diabética, acceso vascular y tiempo de HD ajustado por sexo y edad,

utilizando el programa STATA versión 12.1.

RESULTADOS

En el análisis comparativo de los parámetros hematológicos, el recuento de leucocitos se encontró elevado en los pacientes diabéticos con una diferencia altamente significativa frente a los no diabéticos (p=0.0112). *Ver **Tabla1**.*

Tabla1 .Análisis comparativosde parámetroshematológicos

Parámetros Hematológicos	Diabético (n=11)	No Diabético (n=22)	Valor Normal	Valor p
LEUCOCITOS	6939.6 ±1017.3	5905.5 ±1047.49	5-10000 mm3	**0.011***
NEUTRÓFILOS	62.2 ±9.43	60.3 ±6.83	46-74 und	0.509*
MONOCITOS	8.4 ±1.06	9.1 ±1.59	02-8 und	0.233*
HEMATOCRITO	35.1 ±2.73	36.3 ±3.14	33-46 %	0.289*
HEMOGLOBINA	11.2 ±0.95	11.5 ±1.07	11-16 mg/dl	0.435*
LINFOCITOS	22.9 ±8.85	25.2 ±7.36	20-40 und	0.438*
PLAQUETAS	204.3 ±53.90	194.3 ±66.11	150-450	0.667*
NLR	3.4 ±3.11	2.5 ±1.41	<2	0.380ł
PLR	9.5 ±5.82	7.5 ±3.02	>15	0.380ł
LMR	2.8 ±1.09	2.9 ±0.98	>4	0.802*

Media ± desviación estándar o mediana ± IQR (rangointercuartil)
*Valor dep: *PruebaTstudentyłPruebadeUMann-Whitney*
NLR: Neutrofilo/linfocitoratio,PLR:plaquetas/linfocitoratio,LMR:linfocito/monocitoratio

En el modelo de regresión lineal se aprecia que el LMR disminuye a mayor tiempo de HD, relación estadísticamente significativa (p<0.05). *Ver **Tabla 2 y Figura 1**.*

Tabla 2. Regresión lineal del LMR

	Modelo Crudo			Modelo Ajustado*		
**Variables**	**Coef.**	**IC95%**	**p**	**Coef.**	**IC 95%**	**Valor p**
**Edad**	-0.02	(-0.06-0.02) (	0.238	-0.03	(-0.07-0.02)	0.195
**Género**	0.001	-0.77-0.77)	0.997	-0.35	(-1.18–0.49)	0.403
**Diabético**	0.09	(-0.86-0.67)	0.802	-0.58	(-1.46–0.29)	0.181
**A.vascular**						
CVCLP	R			R		0.369
FAV	-0.2	(-0.95-0.54)	0.583	-0.35	(-1.12–0.43)	
**Tiempo en programa HD (años)**						
(1-3)	R					
(4-6)	-1.09	(-2.12--0.05)	0.041	-1.35	(-2.50--0.20)	**0.023**
>7	-0.69	(-1.63-0.23)	0.134	-1.27	(-2.43--0.11)	**0.034**

R:referencia, Coef. : Coeficiente de la regresiónlineal
Modelo Ajustado: Edad, sexo, si es diabético, acceso vascular y tiempo de hemodiálisis.
LMRp: fue categorizado enLMR alto y bajo considerandolamedia(2.83).

Figura 1.Relación del LMR con tiempo de hemodiálisis (años).

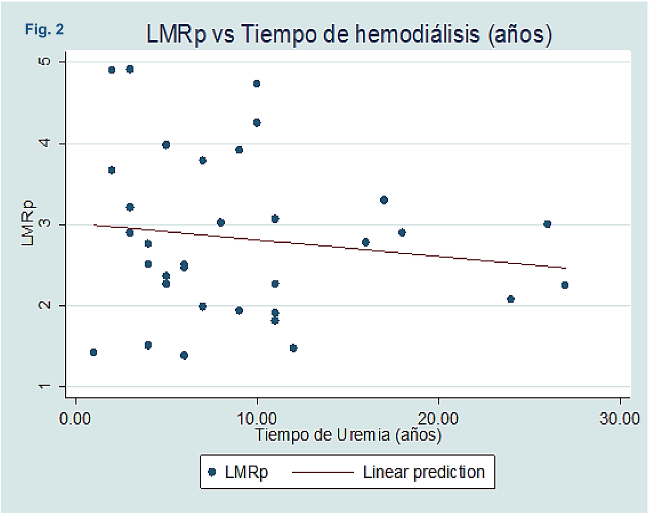

En los parámetros bioquímicos, el nivel de paratohormona fue mayor en los no diabéticos (p=0.047). *Ver **Tabla 3**.*

Tabla 3. Análisis comparativo de parámetros bioquímicos

Parámetros bioquímicos	Diabético (n=11)	No Diabético(n=22)	Valor normal	Valor p
ALBÚMINA	3.9 ±0.13	3.9 ±0.19	3,5-5 g/dl	0.957*
PROTEÍNA C REACTIVA	0.38 ±1.79	0.49 ±1.07	<1 mg/L	0.702ł
CALCIO SÉRICO	9.3 ±0.66	8.9 ±1.02	8,5-10,5 mg/dl	0.309*
HIERRO	9.03 ±4.43	11.23 ±4.96	10,7 -30,4 uM/L	0.380ł
FÓSFORO	5.1 ±0.98	4.5 ±1.49	2,9-5 mg/dl	0.254*
TRANSFERRINA	146.07 ±76.25	156.17 ±39.5	200-400 mg/dl	0.633ł
FERRITINA	371.33 ± 494.04	709.89 ± 436.78	28-365 mg/dl	0.079ł
PARATOHORMONAS	94.65 ± 215.26	226.98 ±216.52	50-300 pg/L	**0.047ł**
KTV	1.7 ± 0.42	1.6 ±0.15		0.529ł
% Saturación de Transferrina(ST)	6.9 ± 2.53	7.8 ± 3.53	20 -50 %	0.439*

Media ±desviación estándaromediana ±IQR (rango intercuartil)
*Valor de p: *Prueba Tstudenty łPrueba de U Mann-Whitney*

DISCUSIÓN

En el perfil hematológico los niveles de leucocitos fueron más altos en los pacientes con DM2, debido a un proceso inflamatorio propio que se sobrepone a la inflamación de la ERC. En relación al nivel de PTH en ambos grupos no existió niveles de Hiperparatiroidismo severo, por lo deducimosque noinfluyecon el LMR.

Este estudio es el primero en nuestro medio en comparar el LMR en pacientes con y sin DM2, en la que no encontramos diferencia significativa, sin embargo, observamos que el LMR disminuye en relación al tiempo de permanencia en HD, hallazgo que podría determinarlaprogresiónde laERC.

CONCLUSIÓN

- El recuento de leucocitos fue mayor en la población diabética, cuyos valoresen ambosgruposestándentrode loreferencial. Este estudio
- muestra que el LMR actúa como un probable marcador proinflamatorio sérico en pacientes con ERC en HD, que es sencillo de medir, de bajo costo y disponible. Potencialmente valioso para darunóptimo seguimientoymanejoclínico.

RECOMENDACIÓN

Se requieren más estudios con un mayor tamaño muestral para incrementar la evidencia científica de estos hallazgos y validar el LMR como un marcador en DM y ERC. Así mismo, se propone evaluar la relación del LMR con las subpoblaciones de monocitos y linfocitos como un elemento de detección de inflamación. A partir de nuestros hallazgos se sugiere evaluar periódicamente el LMR a partir de los 4 años de tratamiento en HD.

Bibliografía:

1.Herrera-Añazco P, Hernández AV, Mezones-Holguin E. Herrera Añazco 2015_ Diabtes y nefropatía en el Perú. Nefrología, Diálisis y Trasplante. 2015;35(4):229 - 37.

2.Zhang J-J, Yang L, Huang J-W, Liu Y-J, Zhang L-X, Zhao M-H, et al. Characteristics and comparison between diabetes mellitus and non-diabetes mellitus among chronic kidney disease patients: A cross-sectional study of the Chinese Cohort Study of Chronic Kidney Disease (C-STRIDE). Oncotarget. 2017;8(63):106324-32.

3.Arrunátegui-Correa V. Despistaje de diabetes mellitus tipo 2 en una población adulta urbana del distrito de Coishco, Ancash,Perú.RevMedHered.2015;26(173-176).

4.Awad AS, You H, Gao T, Cooper TK, Nedospasov SA, Vacher J, et al. Macrophage-derived tumor necrosis factor-alpha mediates diabetic renal injury. Kidney Int. 2015;88(4):722-33.

5.Liakopoulos V, Jeron A, Shah A, Bruder D, Mertens PR, Gorny X. Hemodialysis-related changes in phenotypical features ofmonocytes.Nature.2018;8(13964|).
