# Supplementary material for: Variability of the lymphocyte-to-monocyte ratio in patients with chronic kidney disease on hemodialysis
Source: PLoS One. 2025 Oct 31;20(10):e0333523. doi: 10.1371/journal.pone.0333523 (PMC12578246; doi:10.1371/journal.pone.0333523)
Supplement: Attachment — Submitted filename: Reviewer Letter.docx [file pone.0333523.s003.docx]

**Editor comments**

**Comment E.1**

Two experts raised serious concerns related with the integrity of the current study. They require extensive revision.

**Response E.1**

Dear Editor Tatsuo Shimosawa, M.D., Ph.D., thank you for allowing us to revise our manuscript. In this version, we have replied to all reviewers’ comments and modified the main text and analyses of our study accordingly. Please find all responses below.

**Journal Requirements**

**Comment J.1**

Please ensure that your manuscript meets PLOS ONE's style requirements, including those for file naming. The PLOS ONE style templates can be found at

<https://journals.plos.org/plosone/s/file?id=wjVg/PLOSOne_formatting_sample_main_body.pdf> and

[https://journals.plos.org/plosone/s/file?id=ba62/PLOSOne_formatting_sample_title_authors_affiliations.pdf](https://track.editorialmanager.com/CL0/https:%2F%2Fjournals.plos.org%2Fplosone%2Fs%2Ffile%3Fid=ba62%2FPLOSOne_formatting_sample_title_authors_affiliations.pdf/1/010f0197e321d35f-0a5578eb-e02a-4264-990c-dee6c63a6467-000000/7z8Wk4ZV9ZKOtNhQnrI1gFmB6FEm7AgeXapTiQeB--Q=218)

**Response J.1**

Dear PlosOne team, we have adjusted our manuscript to comply with the journal’s style requirements. Please check the new version of our submission.

**Comment J.2**

We note that your Data Availability Statement is currently as follows: All relevant data are within the manuscript and its Supporting Information files

Please confirm at this time whether or not your submission contains all raw data required to replicate the results of your study. Authors must share the “minimal data set” for their submission. PLOS defines the minimal data set to consist of the data required to replicate all study findings reported in the article, as well as related metadata and methods (<https://journals.plos.org/plosone/s/data-availability#loc-minimal-data-set-definition>).

**Response J.2**

Thank you for the comment. In this new version, we attached the minimal dataset as Supplementary File 1.

**Reviewers comments**

**Reviewer 1**

**Comment 1.1**

General Comments

This retrospective cohort study explores the variability of the lymphocyte-to-monocyte ratio (LMR) in patients with chronic kidney disease (CKD) on hemodialysis and its association with demographic, clinical, and laboratory parameters. The research is timely and relevant, considering the growing interest in inexpensive, accessible inflammatory markers in CKD populations. The study is well-structured and statistically sound, utilizing both cross-sectional and longitudinal regression analyses. The findings are consistent with current knowledge and emphasize the potential role of LMR in inflammation monitoring.

**Response 1.1**

Thank you for this sincere and kind appreciation of our manuscript. In this new version, we have revised all your following comments. We hope to have properly replied to your concerns.

**Comment 1.2**

However, while the study is methodologically adequate, several aspects need clarification or expansion. In particular, further contextualization of LMR among other inflammatory markers (e.g., NLR, PLR, IL-6, TNF-α) would improve the discussion.

**Response 1.2**

Thank you for this relevant comment. In the new version of our manuscript, we have improved the discussion section. Regarding the contextualization of LMR among other inflammatory markers, we included this paragraph:

‘The lymphocyte-to-monocyte ratio (LMR) is an affordable and accessible hematological biomarker. Alsayyad et al. (2019) found that an LMR < 2.66 predicted diabetic nephropathy with high specificity (92%). Similarly, recent studies by Zhou et al. (2024) and Ibrahim (2024) have linked low LMR values with systemic inflammation and progression of chronic kidney disease (CKD).

Although biomarkers such as IL-6 or TNF-α offer greater immunological specificity, their routine use is limited by cost and availability. In contrast, LMR has demonstrated clinical value even in predicting cardiovascular mortality in patients undergoing hemodialysis, with prognostic performance equal to or greater than that of the neutrophil-to-lymphocyte ratio (NLR) and platelet-to-lymphocyte ratio (PLR) (Catabay et al., 2017; Sevecan et al., 2019).

Notably, Xiang et al. (2018) reported that elevated LMR was independently associated with increased all-cause and cardiovascular mortality in hemodialysis patients, outperforming NLR as a prognostic factor. Furthermore, Ibrahim et al. (2024) found that both LMR and IL-6 positively correlated with carotid intima-media thickness, an early marker of atherosclerosis, supporting their utility in predicting disease progression in diabetic nephropathy.

Taken together, these findings underscore the complementary role of LMR as an inflammatory biomarker in CKD. Its inclusion in clinical assessments adds value for both routine monitoring and inflammatory risk stratification. ‘

**Comment 1.3**

Additionally, the use of a single population-based cut-off value for LMR (3.06) could benefit from further justification or sensitivity analysis. Overall, the manuscript presents valuable findings but would benefit from major revision before it is suitable for publication.
**Response 1.3**

Thank you for your valuable observation. In our study, we used a lymphocyte-to-monocyte ratio (LMR) cutoff of 3.06, corresponding to the median value of our study population. This approach is considered valid in exploratory studies when no previously validated threshold is available.

In response to your suggestion, we conducted an additional sensitivity analysis using ROC curve methodology. The results indicated a range of optimal LMR cutoff values between 2.71 and 3.28, which includes the median-derived value of 3.06. Based on these findings, we have included a “Sensitivity Analysis” section in the Methods and Results, and added the following paragraph to the Discussion:

"Although the use of the median is a widely adopted strategy to dichotomize continuous variables (Hui-Ju et al., 2021), this analysis provides new insights by allowing the inclusion of specific LMR cutoff values previously reported in the literature. For instance, Alsayyad et al. (2019) reported an LMR cutoff of 2.66 as an inflammatory marker predictive of diabetic nephropathy development, with a sensitivity of 44% and specificity of 92%. Similarly, Ibrahim et al. (2024) identified a monocyte-to-lymphocyte ratio (MLR) cutoff of 0.3425 associated with chronic kidney disease progression in patients with micro- and macroalbuminuria, which translates to an LMR of 2.92."

**Comment 1.4**

1. While the use of LMR as an inflammatory marker is well-established, the manuscript would benefit from explicitly stating how this study adds to existing literature.
**Response 1.4**

Although the lymphocyte-to-monocyte ratio (LMR) is recognized as a useful inflammatory marker, our study provides novel evidence by analyzing its longitudinal variability in patients with chronic kidney disease (CKD) undergoing hemodialysis. This approach enables assessment of its dynamic behavior over time. Additionally, while using a median-based cutoff value of 3.06, we conducted a sensitivity analysis incorporating alternative thresholds reported in the literature, which yielded consistent results.

We also expanded the discussion by comparing LMR with other inflammatory markers, including the neutrophil-to-lymphocyte ratio (NLR), platelet-to-lymphocyte ratio (PLR), interleukin-6 (IL-6), and tumor necrosis factor-alpha (TNF-α). Previous studies, such as those by Xiang et al. (2018) and Ibrahim et al. (2024), have shown that LMR may be a more robust predictor of mortality and disease progression than other biomarkers, reinforcing its clinical utility and accessibility in resource-limited settings.

**Comment 1.5**

2. The authors used the median LMR value (3.06) as a binary cut-off. While methodologically acceptable in exploratory studies, this lacks external validation.
**Response 1.5**

Thank you for this relevant comment. To strengthen our findings, we have incorporated a sensitivity analysis as commented on in our response 1.3. Unfortunately, we did not identify any publicly available external datasets with complete longitudinal data suitable for additional validation. However, our group is currently compiling a longitudinal dataset of CKD patients undergoing hemodialysis. We expect to complete its organization soon and make it publicly accessible.

**Comment 1.6**

3. The models adjust for major variables such as age, sex, BMI, and diabetes. However, was dialysis adequacy (Kt/V or URR) evaluated?

**Response 1.6**

Thank you for your valuable observation. Indeed, dialysis adequacy is a clinically relevant factor that may influence inflammatory markers and mortality outcomes. Before the current study, we conducted a pilot study in which the average Kt/V in our population was 1.7 ± 0.2. It was well above the minimum recommended threshold of 1.3, which is widely recognized as an indicator of effective hemodialysis treatment. This reduces the likelihood that inadequate clearance influenced inflammatory levels or outcomes related to the LMR (Pérez-García, 2019). Although these results were not included in the present manuscript, they were presented at the 2019 Congress of the Latin American Society of Nephrology and Hypertension (<https://slanh.net/>). The poster can be accessed here:<https://drive.google.com/file/d/1kePxIymcNBXF5G3stwTMin_dmVsxJEd-/view?usp=sharing>.

**References**

- Pérez-García R, Jaldo M, Alcázar R, de Sequera P, Albalate M, Puerta M, et al. El Kt/V alto, a diferencia del Kt, se asocia a mayor mortalidad: importancia de la V baja. Nefrología. 2019;39(1):58–66.

**Comment 1.7**

4. The discussion includes detailed speculation about classical/intermediate/non-classical monocytes. However, the study does not perform monocyte subtyping. Please clarify that this discussion is based on existing literature and not directly measured in the current dataset.

**Response 1.7**

Thank you for this comment. In this new version, we have proofread our discussion to make it less speculative. Please see the corrected text here:

‘Low LMR values are associated with an increased predisposition to developing lymphopenia and/or monocytosis. According to published evidence, this association is particularly linked to the accumulation of senescent monocytes in the context of chronic diseases such as chronic kidney disease (CKD) (26). Monocyte senescence has been associated with telomere shortening, elevated intracellular levels of reactive oxygen species (ROS), and disruption of mitochondrial membrane potential (Zhou et al., 2021). Monocytes can be categorized into classical, intermediate, and non-classical subtypes. Among these, the non-classical proinflammatory monocytes exhibit the most pronounced features of senescence, followed by intermediate and then classical subsets (6).

Additionally, studies have shown that lymphopenia is related to a reduction in regulatory T cells, which are critical for modulating immune responses and preventing tissue damage mediated by inflammation (27). Together, these immunological imbalances contribute to persistent activation of lymphocytes and macrophages, promoting the release of proinflammatory cytokines such as tumor necrosis factor-alpha (TNF-α) and interleukin-6 (IL-6), which directly contribute to glomerular injury and renal fibrosis (6).’

**Reviewer 2**

**Comment 2.1**

This study investigates whether the lymphocyte-to-monocyte ratio (LMR), derived from standard hematological parameters, could serve as a useful inflammatory marker in patients undergoing hemodialysis. Although the topic is clinically relevant and potentially insightful, some important issues remain unresolved.

**Response 2.1**

Thank you for this sincere and kind appreciation of our manuscript. In this new version, we have revised all your following comments. We hope to have properly replied to your concerns.

**Comment 2.2**

Major Points

1. The authors used the median LMR value (3.06) as a cutoff to categorize the hemodialysis patients in this study. It would be helpful to support the decision with citations from previous studies that share a similar focus or clinical relevance.

**Response 2.2**

Thank you for your valuable observation. In our study, we used a lymphocyte-to-monocyte ratio (LMR) cutoff of 3.06, corresponding to the median value of our study population. This approach is considered valid in exploratory studies when no previously validated threshold is available.

In response to your suggestion, we conducted an additional sensitivity analysis using ROC curve methodology. The results indicated a range of optimal LMR cutoff values between 2.71 and 3.28, which includes the median-derived value of 3.06. Based on these findings, we have included a “Sensitivity Analysis” section in the Methods and Results, and added the following paragraph to the Discussion:

"Although the use of the median is a widely adopted strategy to dichotomize continuous variables (Hui-Ju et al., 2021), this analysis provides new insights by allowing the inclusion of specific LMR cutoff values previously reported in the literature. For instance, Alsayyad et al. (2019) reported an LMR cutoff of 2.66 as an inflammatory marker predictive of diabetic nephropathy development, with a sensitivity of 44% and specificity of 92%. Similarly, Ibrahim et al. (2024) identified a monocyte-to-lymphocyte ratio (MLR) cutoff of 0.3425 associated with chronic kidney disease progression in patients with micro- and macroalbuminuria, which translates to an LMR of 2.92."

**Comment 2.3**

2. The manuscript should provide detailed statistics (e.g., mean and distribution) for both the high- and low-LMR groups to allow for a better interpretation of the findings.

**Response 2.3**

Thank you for this comment. We have generated a new Figure 1 to display the full range of LMR values for all patients in this study.

**Comment 2.4**

3. The observation that the type of vascular access may affect the LMR is interesting. Were there any clinical background differences (e.g., age, comorbidities) between the arteriovenous fistula (AVF) and non-AVF groups that could explain the differences in LMR?

**Response 2.4**

Thank you for this important comment. In response to your comment, we looked at potential differences related to Vascular Access with Age (p=0.06), Sex (p=0.5), Etiology (p=0.4), Diabetes mellitus 2 (p>0.9), and Comorbidity (p=0.4).

**Comment 2.5**

4. The association between LMR and sex was noteworthy. Please clarify whether age differences existed between the sexes, whether age influenced the LMR, and whether this sex difference was also present in older individuals, such as postmenopausal women.

**Response 2.5**

Thank you for this insightful comment. In the revised version, we clarified that neither LMR (p = 0.7) nor sex (p = 0.8) was influenced by age in our cohort. Additionally, we assessed the association between age and LMR categories specifically in patients over 45 years old and found no significant relationship (p = 0.6).

**Comment 2.6**

5. Hemoglobin (Hb) and hematocrit (Ht) levels exhibited different trends in relation to LMR. The authors should explore the possible biological or methodological reasons for this discrepancy.

**Response 2.6**

We thank the reviewer for this insightful observation. In response, we have added a discussion of this point in the revised manuscript (Discussion section). Specifically, while both hemoglobin and hematocrit are typically correlated as indicators of red blood cell mass, our longitudinal analysis revealed a positive association between hematocrit and LMR, and a negative association between hemoglobin and LMR.

This apparent discrepancy may reflect distinct physiological or inflammatory dynamics. One plausible explanation is the presence of reactive lymphocytosis during early immune responses or subclinical infections, which may transiently coexist with anemia—a condition commonly observed in CKD patients. Thus, despite lower hemoglobin levels, an increase in lymphocyte count could elevate the LMR. Conversely, hematocrit, being more stable and less sensitive to short-term fluctuations, may better reflect the underlying chronic inflammatory burden. This differentiation highlights the complex immuno-hematologic landscape in hemodialysis patients and underscores the need for future studies with more refined immunophenotyping to fully elucidate these patterns.

**Comment 2.7**

6. The manuscript frequently cites studies investigating the monocyte-to-lymphocyte ratio (MLR), although the present study focused on the LMR instead. As these two markers are mathematically inverse and can differ in clinical interpretation, the authors should clarify whether the findings from MLR studies can be extrapolated to LMR. Additionally, the rationale for selecting LMR over MLR in this study should be discussed.

**Response 2.7**

Thank you for your observation. We clarify that this study utilizes the lymphocyte-to-monocyte ratio (LMR), rather than its inverse (monocyte-to-lymphocyte ratio, MLR), as the clinical interpretation of these indices may vary depending on the context. The choice of LMR reflects its widespread use in nephrology and other chronic conditions, such as cancer, where it is employed to represent immunological balance: a low LMR indicates systemic inflammation, characterized by a reduced proportion of lymphocytes relative to monocytes (Alsayyad, 2019; Pan, 2018).

We acknowledge, however, that several relevant studies use MLR as the primary marker. Given that both indices are derived from the same cellular components and may be interchangeable under certain epidemiological conditions, findings based on MLR can be considered complementary when interpreting LMR. As noted by Zhou et al. (2024) and Ibrahim et al. (2024), elevated MLR values (and thus low LMR) are associated with increased inflammation and CKD progression. This inverse relationship has been consistently observed in our review of the literature.

Therefore, while our primary focus on LMR is based on its interpretative clarity and methodological precedent, we have incorporated relevant MLR-based studies in the discussion to enrich the conceptual framework and facilitate comparison with existing literature.

**References**

- Alsayyad M, Abd H. The prognostic value of lymphocyte-to-monocyte ratio in nephropathy of type 2 diabetes mellitus. Sci J Al-Azhar Med Fac Girls. 2019;3(1):181.
- Ibrahim, H. M. M., Bahgat, H. M., Sharshar, D. A., & Ramzy, T. A. A. (2024). Monocyte lymphocyte ratio, IL 6, and their association with increased carotid intima-media thickness as simple predictive markers for nephropathy in Egyptian diabetic patients. The Egyptian Journal of Internal Medicine. https://doi.org/10.1186/s43162-024-00284-x
- Pan YC, Jia ZF, Cao DH, Wu YH, Jiang J, Wen SM, et al. Preoperative lymphocyte-to-monocyte ratio (LMR) could independently predict overall survival of resectable gastric cancer patients. Medicine (Baltimore). 2018;97(52):1–7.
- Zhou, Q., Shao, X., Li, X., Zou, H., & Chen, W. (2024). Association between Monocyte-to-Lymphocyte Ratio and Inflammation in Chronic Kidney Disease : A Cross-Sectional Study. Kidney & Blood Pressure Research, 1–19.<https://doi.org/10.1159/000542625>

**Comment 2.8**

7. Several references cited as supporting LMR actually discuss MLR (e.g., references 8, 9, 15, 18, 28, 29, and 43). To ensure clarity, please specify whether each reference pertains to LMR or MLR.

**Response 2.8**

Thank you for this important observation. In this new version, we commented on every mention of LMR or MLR, which was the index in discussion.

**Comment 2.9**

Minor Points

1. Table 2: The high-LMR group included 59 patients, but the number of cases listed under CKD etiology did not add up to this total. Please review and revise the case counts and their respective percentages accordingly.

**Response 2.9**

We apologize for this involuntary error. We identified a mistake in the etiology distribution and have now verified and corrected the values accordingly. The updated data are presented in the revised version (see Table 2).

**Comment 2.10**

2. Table 4: The use of "TGP" and "TGO" is unnecessary as these are non-standard terms. It is sufficient to report liver enzymes as "AST" and "ALT.” In addition, because "Uric Acid" was not measured, it should be removed from the table.

**Response 2.10**

Thank you for this comment. We have modified our manuscript accordingly with your suggestions.

**Comment 2.11**

3. Page 6, Lines 26–29: Reference 15 relates to MLR and CKD onset, not aging. References 6 and 16 also did not discuss LMR specifically. These references should be reviewed and, if necessary, replaced with more appropriate ones.

**Response 2.11**

Thank you for this comment. Related to the reference 15, a direct relation between aging and MLR in chronic kidney disease was reported. The article textually expresses: *“In addition, supplemental table 1 showed the association between MLR and other variables. MLR was positively related to inflammatory factors, such as leukocytes, neutrophils, NLR, PLR and platelet distribution width. In addition, MLR was positively correlated with age, blood pressure and BMI. However, there was no significant correlation between MLR and fasting plasma glucose in non-diabetic participants.”*. Regarding other references, we have extensively accurate the descriptions of all cited references.

**Comment 2.12**

4. Page 16, Lines 23–31: Reference 44 by Valga et al. (2020) focused on erythropoietin resistance and did not support the conclusions regarding PLR/NLR as predictors of mortality or cardiovascular events. This reference should be revised or removed.

**Response 2.12**

Thank you for the observation. The reference to Valga has been removed and replaced with other appropriate related references, accompanied by the following text:

‘It is worth noting that other inflammatory biomarkers such as neutrophil-to-lymphocyte ratio (NLR), platelet-to-lymphocyte ratio (PLR), and LMR have shown predictive value for severe complications in patients with diabetic foot, including peripheral artery disease, osteomyelitis, and risk of amputation (Dermidal, 2018). Moreover, available evidence supports that MLR, the inverse of LMR, is a more consistent and predictive inflammatory marker than NLR and PLR in various clinical contexts. In patients with acute hemorrhagic stroke, MLR was significantly associated with in-hospital mortality, while NLR showed limited predictive power (Jiang, 2022). In CKD, MLR was not only positively correlated with inflammation, disease progression, and mortality risk, but also outperformed other indicators such as NLR, TNF-α, CRP, and estimated glomerular filtration rate (eGFR) (Zhou, 2024; Sari, 2024; Liu, 2024).’
