# Supplementary material for: Variability of the lymphocyte-to-monocyte ratio in patients with chronic kidney disease on hemodialysis
Source: PLoS One. 2025 Oct 31;20(10):e0333523. doi: 10.1371/journal.pone.0333523 (PMC12578246; doi:10.1371/journal.pone.0333523)
Supplement: Attachment — Submitted filename: Reviewer Letter (1).docx [file pone.0333523.s004.docx]

**Editor comments**

**Comment E.1**

There still remain some minor pionts to be rivised.

**Response E.1**

Dear Editor Tatsuo Shimosawa, M.D., Ph.D., thank you for allowing us to revise our manuscript. In this version, we have replied to all reviewers’ comments and modified the main text and analyses of our study accordingly. Please find all responses below.

**Journal Requirements**

**Comment J.1**

1. If the reviewer comments include a recommendation to cite specific previously published works, please review and evaluate these publications to determine whether they are relevant and should be cited. There is no requirement to cite these works unless the editor has indicated otherwise.

**Response J.1**

Dear PlosOne team, Thank you for this advice. In this version, we have revised all suggestions to include and revise references to make proper decisions.

**Comment J.2**

2. Please review your reference list to ensure that it is complete and correct. If you have cited papers that have been retracted, please include the rationale for doing so in the manuscript text, or remove these references and replace them with relevant current references. Any changes to the reference list should be mentioned in the rebuttal letter that accompanies your revised manuscript. If you need to cite a retracted article, indicate the article’s retracted status in the References list and also include a citation and full reference for the retraction notice.

**Response J.2**

Thank you for the comment. In this new version, we have revised all references and changed some of them. All changes are highlighted in the track_changes file.

**Reviewer 1**

**Comment 1.1**

The authors have responded to most of my initial comments in a constructive and detailed manner. I appreciate the substantial improvements, including the addition of a sensitivity analysis for the LMR cut-off, expanded discussion comparing LMR with other inflammatory markers, and corrections to tables and terminology. The manuscript is now clearer and more informative. However, several important issues remain insufficiently addressed. These points should be resolved before the manuscript can be considered ready for publication.

**Response 1.1**

Thank you for the comment. In this new version, we have included improvements to attend to your comments. We hope our answers are aligned with your requirements.

**Comment 1.2**

While this study focuses on LMR, many cited studies investigated the monocyte-to-lymphocyte ratio (MLR). Although the authors note that LMR and MLR are mathematically related, their clinical interpretation and predictive ability are not always interchangeable. Please explicitly indicate in the text (or ideally in a summary table) whether each cited reference pertains to LMR or MLR. Clarify the limits of extrapolating findings from MLR to LMR and avoid implying full equivalence in prognostic implications.

**Response 1.2**

Thank you for the comment. In this new version, we modified lines 390-395 to comment on the limitations of extrapolating data based on LMR or MLR. In addition, we summarised all relevant information for MLR or LMR in Table 7.

**Comment 1.3**

The addition of ROC-based sensitivity analysis is commendable. However, the analysis was performed within the same dataset, limiting external generalizability. Please explicitly state in the Discussion that the median-derived cut-off (3.06) remains exploratory and requires validation in independent cohorts before it can be recommended for clinical use.

**Response 1.3**

Thank you for the comment. In this new version, we added the following text: *‘In addition, it is important to point out that the cutoff derived from the median in our study (3.06) remains exploratory and requires validation in independent cohort studies before it can be recommended for clinical use.’ (page 15)*

**Comment 1.4**

The adequacy of dialysis (Kt/V, URR) is an important confounder that may influence inflammatory markers and outcomes. The authors refer to a pilot study, but no adequacy data for the current study population are presented. If available, please provide actual Kt/V or URR values for the study cohort and consider including them in multivariate models. If not available, explicitly acknowledge this as a limitation.

**Response 1.4**

Thank you for the comment. In this new version, we added the following paragraph on page 19:

*‘Moreover, dialysis adequacy (Kt/V or URR) is a relevant confounding factor, as it may influence both inflammatory markers and clinical outcomes. In our cohort, these data were not available and thus could not be included in the multivariate adjustment, which represents a limitation of the study. However, in a prior pilot study conducted in our population, the average Kt/V was 1.7 ± 0.2 (*[*https://n9.cl/mv2k2*](https://n9.cl/mv2k2)*, S2 File), well above the minimum recommended threshold of 1.3 (Pérez-García, 2019), which is widely recognized as an indicator of effective hemodialysis. This finding reduces the likelihood that inadequate clearance influenced inflammatory levels or outcomes related to the LMR.’*

**Comment 1.5**

The section on monocyte subpopulations remains largely speculative, as no direct measurements were performed. Please clearly state in the Discussion that these mechanistic interpretations are based on literature, not on data from the current study.

**Response 1.5**

Thank you for the comment. In this new version, we added the following text between lines 368-371:

*‘It is important to note that while monocyte and lymphocyte subpopulations were not directly measured in this study, the following mechanistic interpretations are based on previously published literature.’*

**Comment 1.6**

The study’s main novelty is the longitudinal evaluation of LMR variability. However, the practical implications for patient management and prognosis remain underdeveloped. Please expand on how monitoring longitudinal LMR might alter clinical decision-making or improve risk stratification in hemodialysis patients.

**Response 1.6**

Thank you for the comment. In this new version, we added the following text on page 19:

*‘The findings of this study reinforce the clinical value of the LMR as an indicator of the inflammatory state in patients with chronic kidney disease. This suggests that longitudinal monitoring of LMR could serve as a simple and accessible tool for the dynamic stratification of risk in hemodialysis. Serial assessments would enable the identification of patients with persistent or progressive inflammation, facilitate the prediction of cardiovascular complications and mortality, and guide targeted interventions aimed at optimizing dialysis adequacy, improving anemia management, and preventing infections. The routine integration of LMR into clinical practice could therefore promote more personalized decision-making and the early identification of patients at higher risk.’*

**Reviewer 2**

**Comment 2.1**

The author has responded carefully and appropriately to the previous reviewers' comments. An additional analysis using the ROC curve to explore the relationship between LMR and other inflammatory markers (such as NLR and PLR) is a valuable addition to the study. While the content and structure show improvement, I still have significant concerns regarding the accuracy and appropriateness of references. Please consider the following points for further revision:

**Response 2.1**

Thank you for the comment. In this new version, we have included improvements to attend to your comments. We hope our answers are aligned with your requirements.

**Comment 2.2**

New Figure 1: The figure uses both gray and black dots. Please add an explanation in the figure caption to clarify what each type of dot represents.

**Response 2.2**

Thank you for the comment. In this new version, we complemented the legend of Figure 1 to clarify the meaning of these different grey/black tones. They are related to data density.

*‘****Fig 1. Lymphocyte to Monocyte ratio from the cohort of this study.*** *We plotted all captured levels for LMR of these patients with a maximum follow-up time of 35.5 months. Each grey dot represents an individual patient measurement, the black dots denoting values within the central density of the data distribution for better visualization. The dashed red line marks the baseline median LMR (3.06).’*

**Comment 2.3**

Page 5, line 5: Reference 7 has been changed from the previous version to the current version. Please verify its appropriateness and reconsider whether reference 6 is still necessary in this context.

**Response 2.3**

Thank you for the comment. We realized an involuntary mistake, now been corrected.

References 6-7:

6.	Ong S-M, Hadadi E, Dang T-M, Yeap W-H, Tan CT-Y, Ng T-P, et al. The pro-inflammatory phenotype of the human non-classical monocyte subset is attributed to senescence. Cell Death Dis. 2018;9: 266. doi:10.1038/s41419-018-0327-1

7.	Xia Y, Yang Q, Wu SY, Wu Z, Li Q, Du J. Interferon lambda modulates proinflammatory cytokines production in PBMCs from patients with chronic kidney disease. Hum Immunol. 2023;84: 464–470. doi:10.1016/j.humimm.2023.06.001

**Comment 2.4**

Page 5, lines 31-32: Please re-check reference 23, as it does not appear to discuss CKD progression.

**Response 2.4**

Thank you for the comment. We apologize for this mistake. We corrected this reference for Sari et al., 2024.
 Sari D, Wardhani P, Puspitasari Y, Suryantoro S. Association of monocyte-to-lymphocyte ratio, neutrophil-to-lymphocyte ratio, and tumor necrosis factor-α in various stages of chronic kidney disease. Journal of Advanced Biotechnology and Experimental Therapeutics. 2024;7: 346. doi:10.5455/jabet.2024.d29

**Comment 2.5**

Page 6, line 3: Please confirm that reference 10 is appropriate, as it does not primarily focus on peritoneal dialysis patients.

**Response 2.5**

Thank you for the comment. We updated the description of that reference to align with their content.

*‘’Zhou et al. (2024) demonstrated that MLR is a simple and low-cost biomarker that reflects systemic inflammation and helps physicians in the management of nephropathies [10]. ‘*

**Comment 2.6**

Page 15, line 6: Please confirm that reference 21 is a suitable citation, as it does not include hemodialysis patients.

**Response 2.6**

Thank you for the comment. Indeed, the referenced study corresponds to a prospective cohort of 1,280 participants in China, followed for nearly two years, in which the variability of the MLR and its association with the development of chronic kidney disease (CKD) were analyzed.

The change is made to: *“Conversely, elevated MLR values, reflecting lower LMR, have been linked to aging [21], increased cardiovascular risk [22], CKD progression [10,23], and high mortality due to CKD [22].”*
